# Supplementary material for: Cryo-EM and femtosecond spectroscopic studies provide mechanistic insight into the energy transfer in CpcL-phycobilisomes
Source: Nat Commun. 2023 Jul 5;14:3961. doi: 10.1038/s41467-023-39689-7 (PMC10322944; doi:10.1038/s41467-023-39689-7)
Supplement: Supplementary file 1 — Supplementary Information [file 41467_2023_39689_MOESM1_ESM.pdf]

Cryo-EM and femtosecond spectroscopic studies provide mechanistic insight into the energy transfer in CpcL-phycobilisomes

Lvqin Zheng[1,2], Zhengdong Zhang[1,3], Hongrui Wang[1,3], Zhenggao Zheng[1,3], Jiayu Wang[4], Heyuan Liu[4], Hailong Chen[4], Chunxia Dong[1,3], Guopeng Wang[1], Yuxiang Weng[4,*], Ning Gao[1,2,*] & Jindong Zhao[1,3,*]

[1]School of Life Sciences, Peking University, Beijing 100871, China

[2]State Key Laboratory of Membrane Biology, Peking University, Beijing 100871, China

[3]State Key Laboratory of Protein and Plant Gene Research, Peking University, Beijing 100871, China

[4]Laboratory of Soft Matter Physics, Institute of Physics, Chinese Academy of Sciences, Beijing 100190, China

These authors contributed to this work equally: Lvqin Zheng, Zhengdong Zhang, Hongrui Wang, Zhenggao Zheng

*Corresponding authors, Y.W. yxweng@iphy.ac.cn; N.G. gaon@pku.edu.cn; J.Z. jzhao@pku.edu.cn

Supplementary Figures

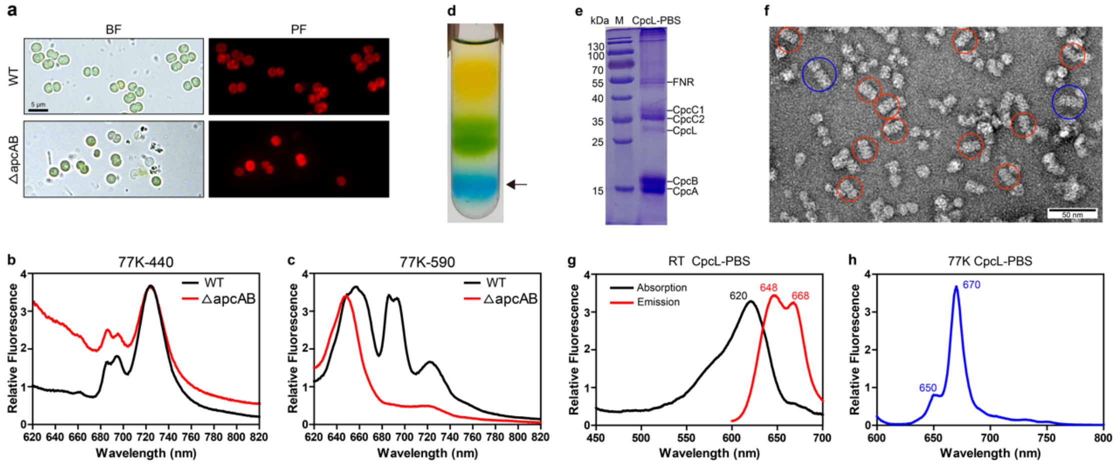

Supplementary Fig. 1 : Preparation and characterization of CpcL-PBS from *Synechocystis* 6803.

a Images of the wild-type *Synechocystis* 6803 cells (upper panels) and the cells of the deletion mutant of *apcBA* (ΔapcAB, lower panels) in bright field (BF) and pigment fluorescence (PF). Experiments were repeated more than three times with similar results.

b 77 K fluorescence emission spectra of the wild-type (black) and ΔapcAB (red) excited with a 440 nm light.

c 77 K fluorescence emission spectra of the wild-type (black) and ΔapcAB (red) excited with a 590 nm light.

d Image of sucrose gradient ultracentrifugation. The blue band after centrifugation as indicated by the arrow contained CpcL-PBS.

e SDS-PAGE analysis of CpcL-PBS. The gel was stained with Coomassie brilliant blue. Left lane, molecular marker standards and the molecular masses in kDa are indicated on the left. Right lane, CpcL-PBS components, the identified proteins are labeled on the right side of the gel. Experiments were repeated more than three times with similar results.

f Electron microscopic images of negatively stained CpcL-PBS particles. Particles with three layers and more than three layers of αβ hexamers are highlighted in red and blue circles, respectively. Experiments were repeated more than three times with similar results. Scale bar indicates 50 nm.

g Room temperature absorption spectrum (black) and fluorescence spectrum of CpcL-PBS (red). Fluorescence spectrum was obtained by excitation with a 590 nm light. The numbers indicate the wavelengths of the absorption or emission peaks.

h 77K fluorescence spectrum of CpcL-PBS excited with a 590 nm light.

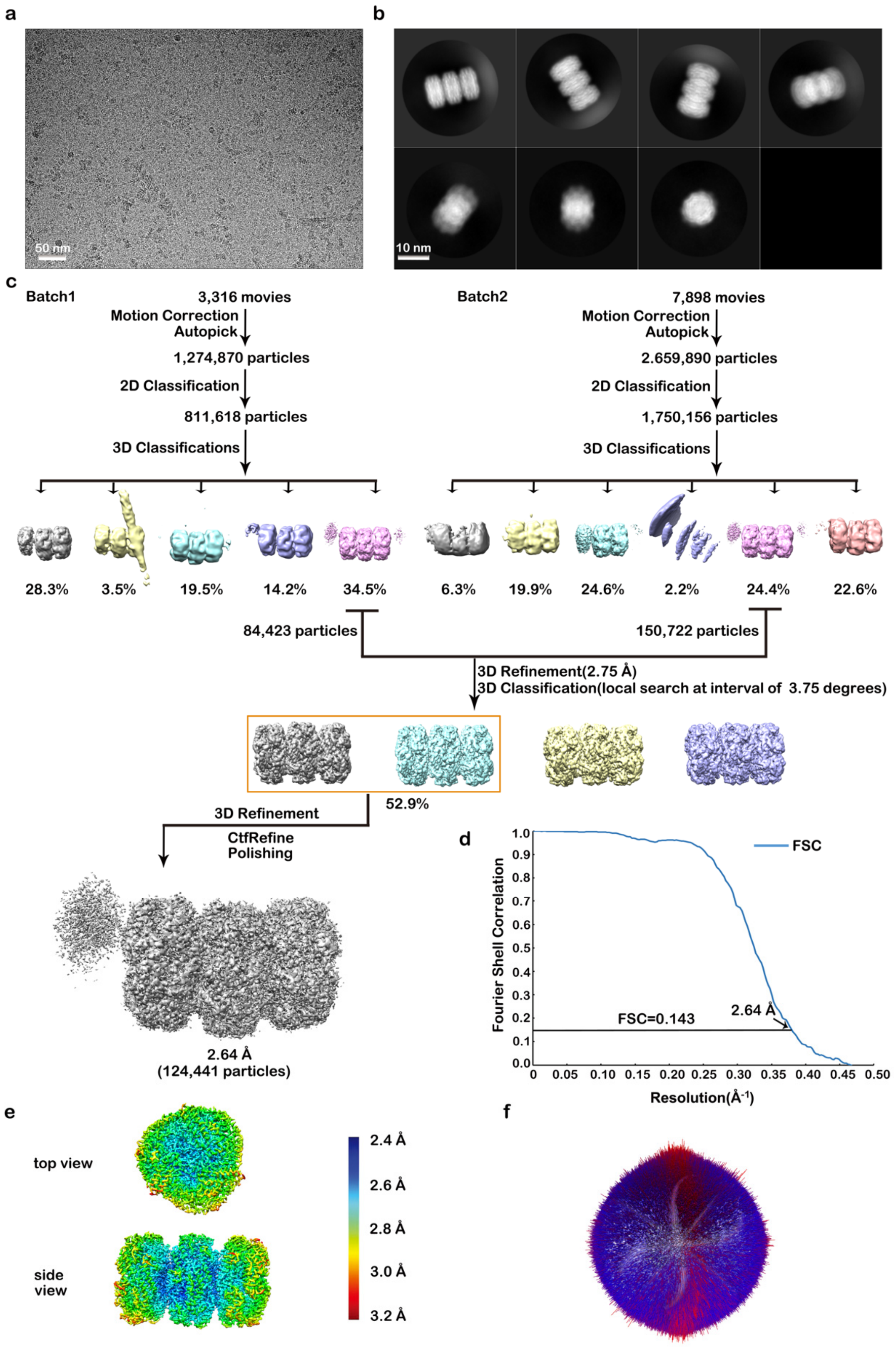

Supplementary Fig. 2 : Workflow of the cryo-EM image processing.

a A representative raw cryo-EM image. Experiments were repeated more than three times with similar results.

b Representative 2D class averages of the CpcL-PBS particles from *Synechocystis* 6803.

c Image processing workflow, including 3D classification, structural refinement, masked-based refinement, CTF refinement and Bayesian polishing.

d Gold-standard Fourier shell correlation (FSC) of the final cryo-EM map.

e Local resolution estimation of the final density map.

f Angular distribution of the CpcL-PBS particles in the final round of 3D refinement.

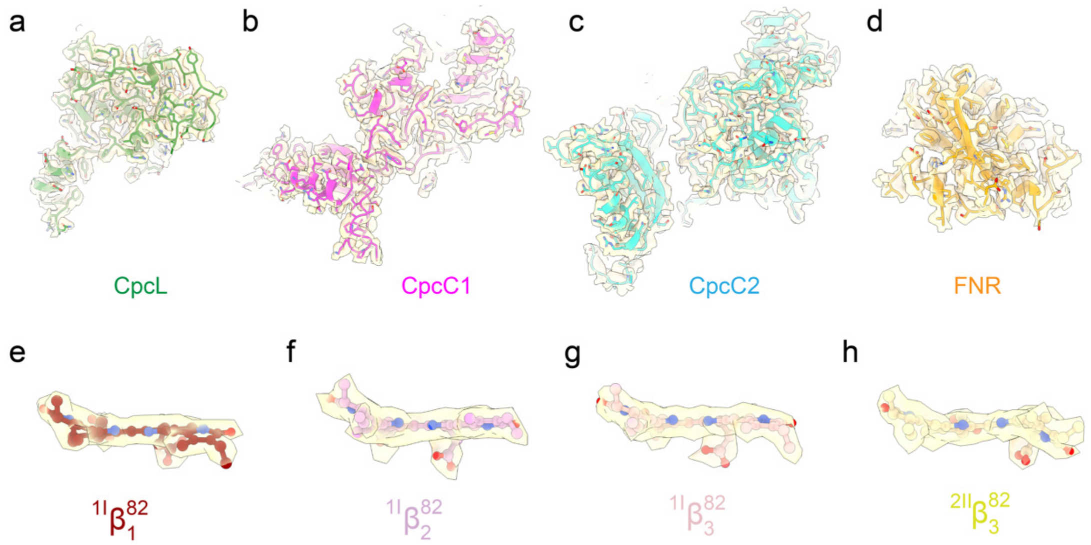

Supplementary Fig. 3 : Local densities of representative PBS subunits and bilins in CpcL-PBS.

a-d Representative PBS subunits in CpcL-PBS, CpcL **(a)**, CpcC1 **(b),** CpcC2 **(c)** and FNR **(d)**.

e-h Representative bilins in CpcL-PBS, bilin $^{1I}\beta^{82}{}_{1}$ **(e)**, bilin $^{1I}\beta^{82}{}_{2}$ **(f)**, bilin $^{1I}\beta^{82}{}_{3}$ **(g)** and bilin $^{2II}\beta^{82}{}_{3}$ **(h)**.

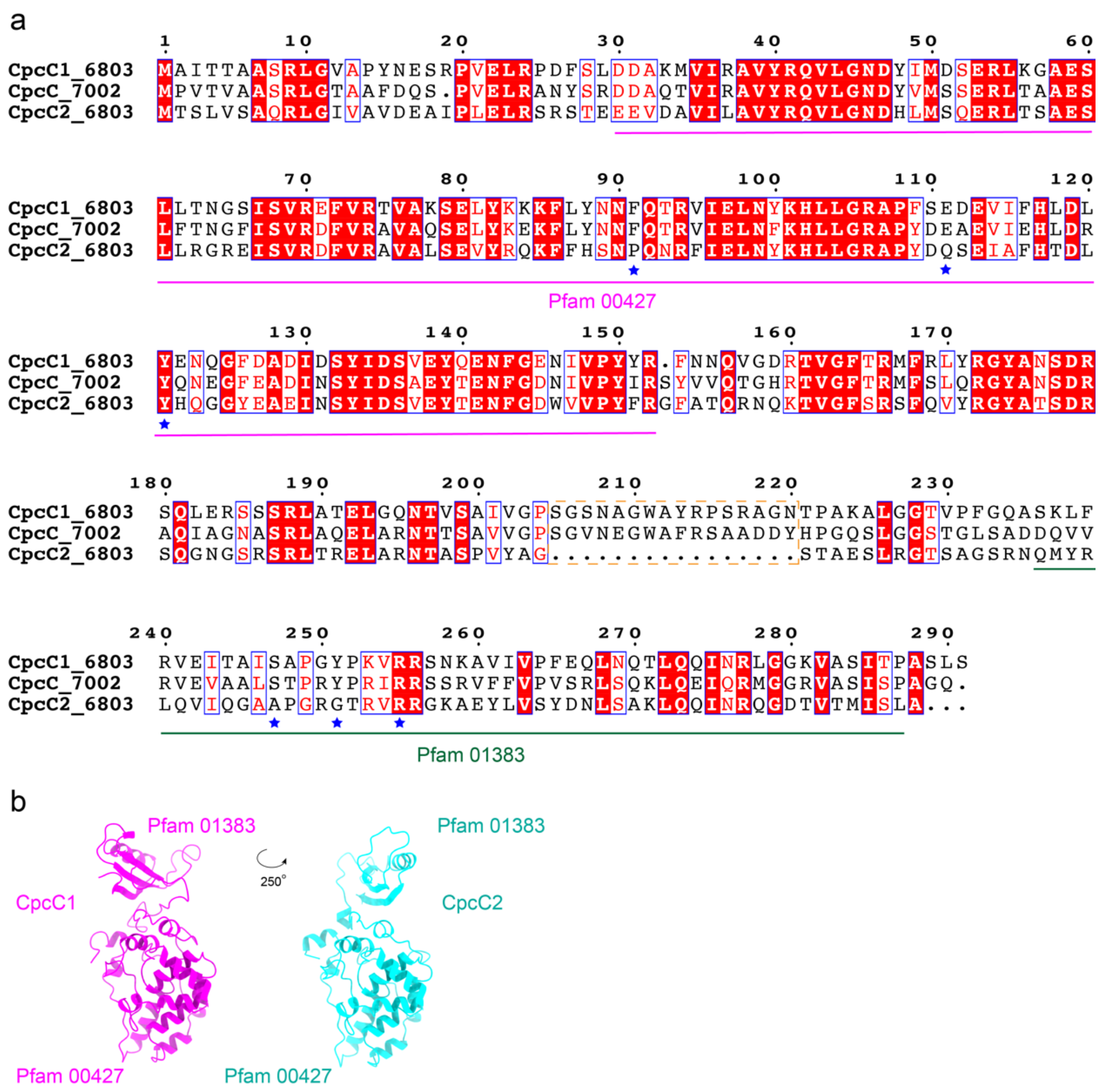

Supplementary Fig. 4 : Sequence alignment of CpcC proteins and structural alignment of CpcC1 and CpcC2.

a Alignment of CpcC1, CpcC2 sequences from *Synechocystis* 6803 and CpcC sequence from *Synechococcus* 7002. The sequence is numbered according to the CpcC1 sequence of *Synechocystis* 6803. The interacting residues with bilins in CpcC1 are highlighted with blue pentagrams. The major difference between CpcC1 and CpcC1 proteins from *Synechocystis* 6803 are highlighted in orange dash-line boxes. The Pfam 00427 and Pfam 01383 domains of CpcC1 are highlighted in magenta and forest green solid lines, respectively.

b Alignment of CpcC1 and CpcC2 structures from *Synechocystis* 6803.

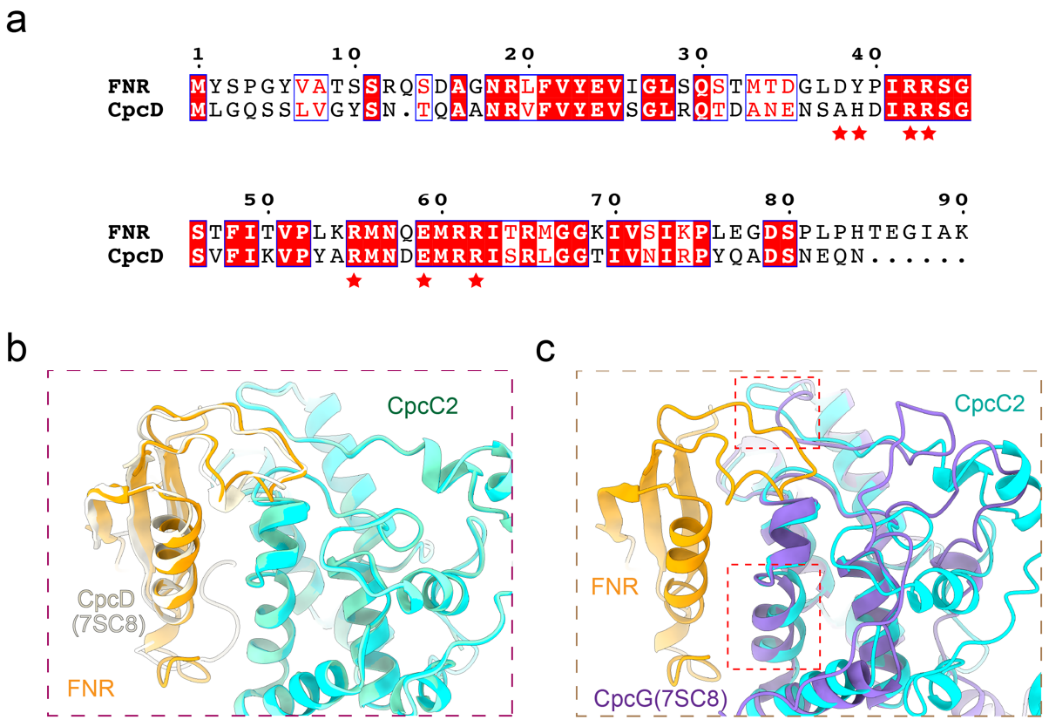

Supplementary Fig. 5 : Structure and sequence analysis of FNR.

a Sequence alignment of CpcD and CpcD-like domain of FNR. Residues that interact with CpcC2 are highlighted with red pentagrams.

b Structural comparison of FNR and CpcD reveals a similar binding mode with CpcC2.

c Structural superimposition of CpcG onto the structure of FNR-CpcC2, showing a potentially a distinct binding mode with FNR. The different regions in binding surface are circled with red dashed rectangle. The structures of CpcD and CpcG are adopted from the rod of CpcG-PBS from *Synechocystis* 6803 (PDB: 7SC8).

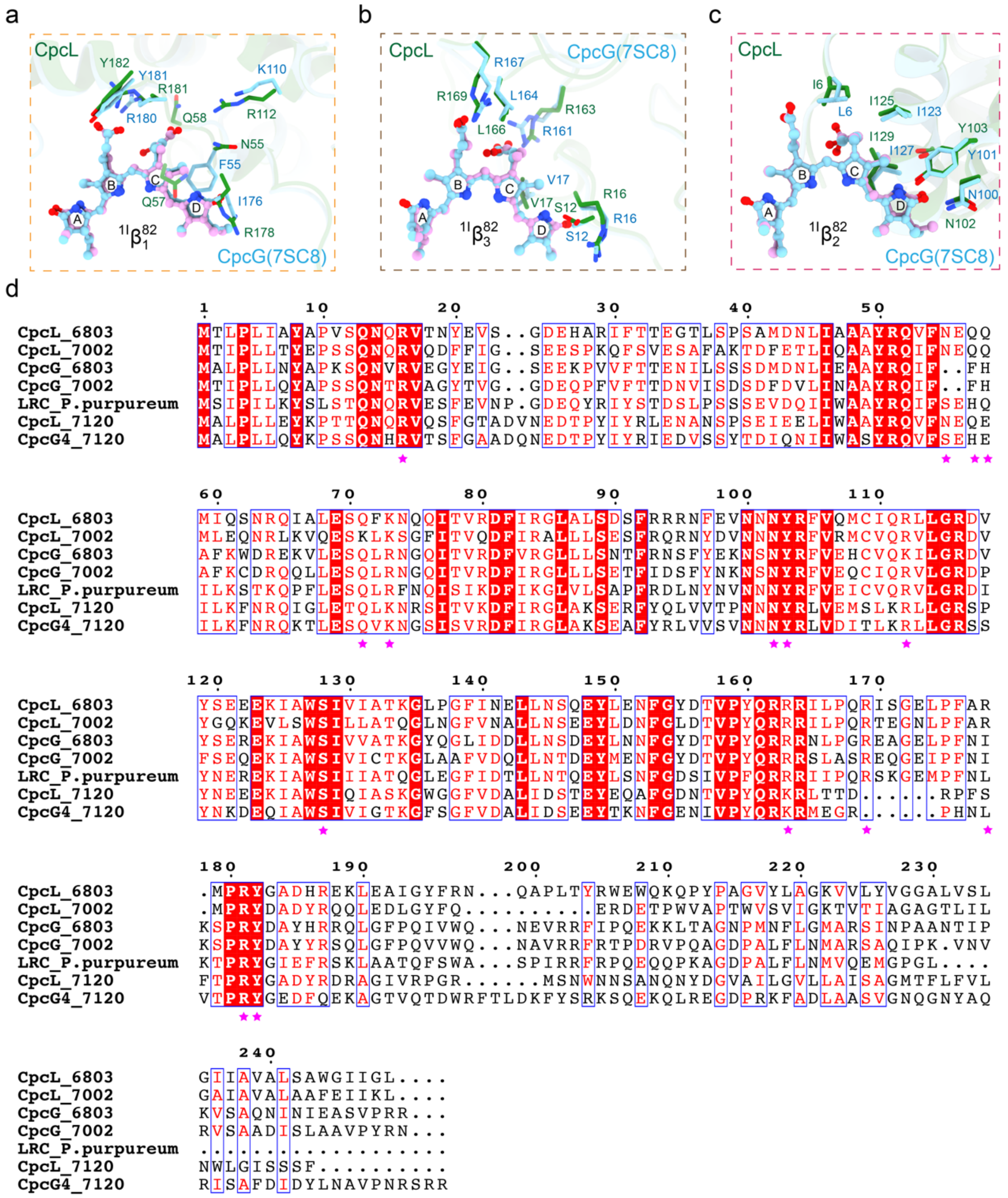

Supplementary Fig. 6 : Structural comparison and sequence alignment of CpcL and CpcG.

a-c Structural comparison of the surrounding residues of bilin $^{1I}β^{82}_1$ (**a**), bilin $^{1I}β^{82}_3$ (**b**) and bilin $^{1I}β^{82}_2$ (**c**) from CpcG-PBS rod and CpcL-PBS.

d Alignment of CpcL, CpcG sequences from *Synechocystis* 6803, *Synechococcus 7002*, *Anabaena 7120* and *Porphyridium purpureum*. The sequence is numbered according to the CpcL sequence of *Synechocystis* 6803. The interacting residues with bilins in CpcL

are highlighted with magenta pentagrams.

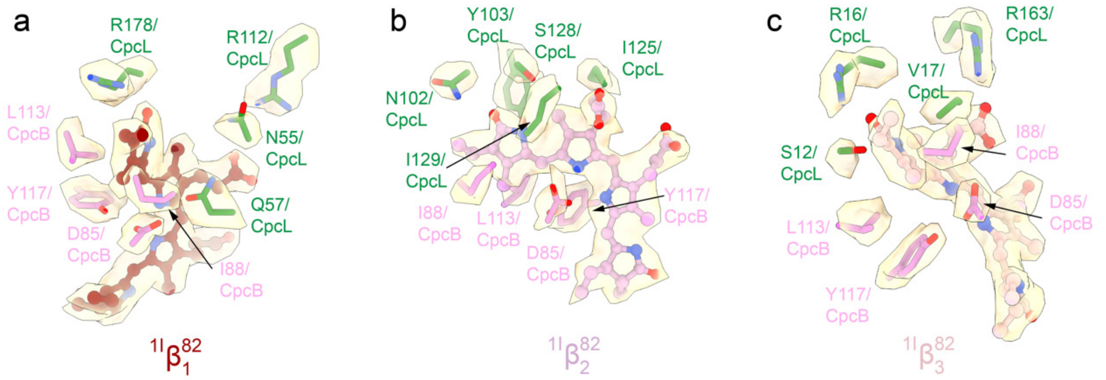

Supplementary Fig. 7 : Local environment of ring D of the bilin $^{1I}\beta^{82}{}_1$, $^{1I}\beta^{82}{}_2$ and $^{1I}\beta^{82}{}_3$ from *Synechocystis* 6803.

a-c Local environment of the ring D of the bilins $^{1I}\beta^{82}{}_1$ (**a**), $^{1I}\beta^{82}{}_2$ (**b**) and $^{1I}\beta^{82}{}_3$ (**c**). Residues from CpcL is colored as forest green and the CpcB is colored as pink, respectively. Bilins are color-coded same as Fig. 3c.

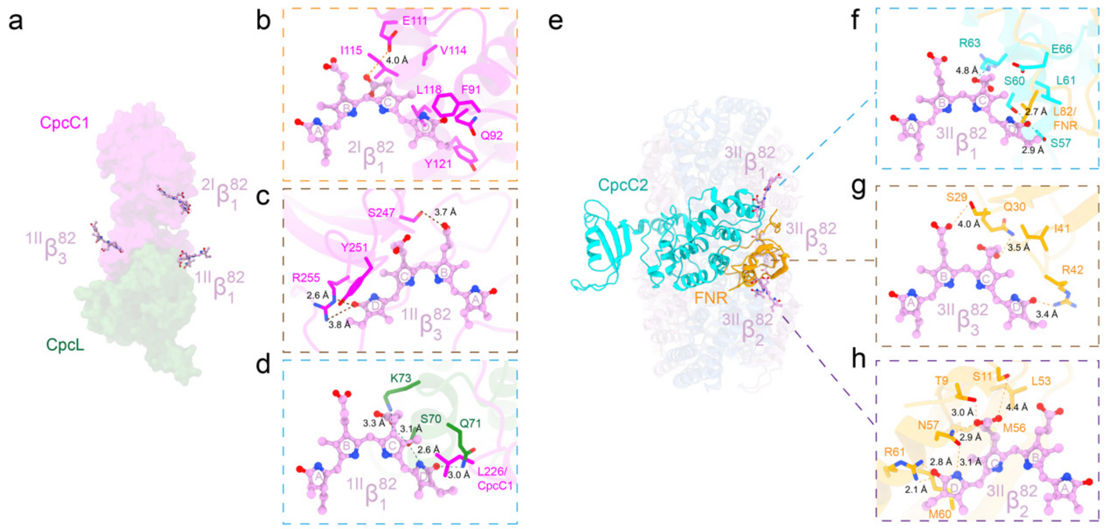

Supplementary Fig. 8 : Interactions of CpcC1 and FNR with chromophores.

a Side view of CpcL and CpcC1 structures. The CpcL and CpcC1 are shown in surface representation. Three special bilins are shown in a ball-stick representation.

b-c The surrounding residues of bilins $^{2I}\beta^{82}{}_1$ and $^{1II}\beta^{82}{}_3$. These two bilins adopt a flatted conformation in their ring D. The polar interactions are highlighted with blank dashed lines.

d, The surrounding residues of bilins $^{1II}\beta^{82}{}_1$. Both the CpcL and CpcC1 are involved in the interactions.

e, Overall view of the interactions between FNR and its surrounding bilins.

f-h, Interaction details of the bilins and the interacting residues in CpcC2 and FNR. The surrounding residues of bilins $^{1II}\beta^{82}{}_1$ are shown and their color is same as Figure 1.

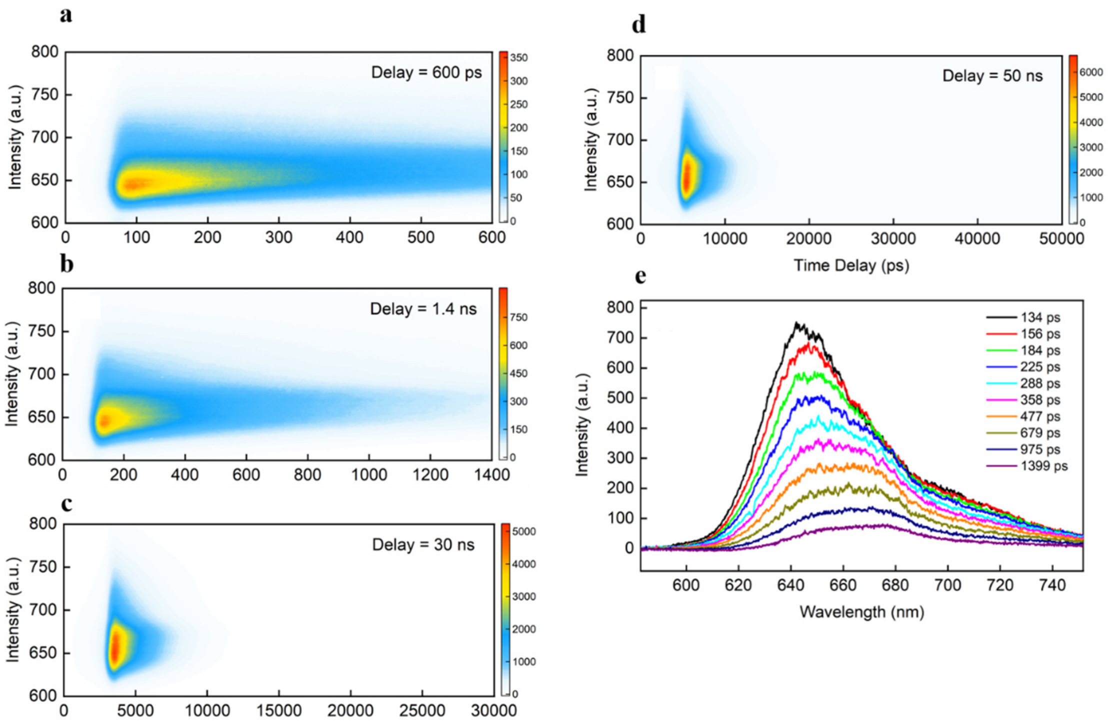

Supplementary Fig. 9 : Ultrafast fluorescence spectroscopic measurement of CpcL-PBS energy transfer.

a-d Time-wavelength 2D maps with various delay times indicated in each panel. Data were collected with picosecond streak camera.

e Time-resolved fluorescence spectra of CpcL-PBS excited at 565 nm. The delay times of each spectrum are shown in the panel with respective colors.

Supplementary Tables

Supplementary Table 1. Sequences of the oligonucleotides used as primers in this study

Primer name	Nucleotide sequence
P1	GCGGTGTAAGGGGGGGTAAAG
P2	gtataattatagcacgcgGGATGGATTCCTCCGTAAAG
P3	cgatgataagctgtcaaacatgagTCCTGGATTCCCGTGGGTGATG
P4	CCTAGATCCTGGTGTGGCCTTTG
P5	CTTTACGGAGGAATCCATCCcgcgtgctataattatactaat
P6	CATCACCCACGGGAATCCAGGActcatgtttgacagcttatcatcg

Supplementary Table 2. Cryo-EM data collection, refinement and validation statistics

	CpcL-PBS-FNR (EMDB-34724) (PDB 8HFQ)
**Data collection and processing**	
Magnification	81,000
Voltage (kV)	300
Electron exposure (e–/Å^2)	60
Defocus range (μm)	-1.0 to -1.8
Pixel size (Å)	1.07
Symmetry imposed	C1
Initial particle images (no.)	3.934,760
Final particle images (no.)	124,441
Map resolution (Å)	2.64
FSC threshold	0.143
Map resolution range (Å)	2.0-3.6
**Refinement**	
Initial model used (PDB code)	4F0T
Model resolution (Å)	2.64
FSC threshold	0.143
Map sharpening *B* factor (Å^2)	-42.14
Model composition	
Non-hydrogen atoms	54,217
Protein residues	6,851
Ligands	54
B factors (Å^2)	
Protein	29.47
Ligand	29.28
R.m.s. deviations	
Bond lengths (Å)	1.196
Bond angles (°)	0.008
Validation	
MolProbity score	1.43
Clashscore	7.96
Poor rotamers (%)	0.00
Ramachandran plot	
Favored (%)	98.30
Allowed (%)	1.70
Disallowed (%)	0.00
