# Supplementary material for: Cryo-EM and femtosecond spectroscopic studies provide mechanistic insight into the energy transfer in CpcL-phycobilisomes
Source: Nat Commun. 2023 Jul 5;14:3961. doi: 10.1038/s41467-023-39689-7 (PMC10322944; doi:10.1038/s41467-023-39689-7)
Supplement: Supplementary file 4 — Source Data [file 41467_2023_39689_MOESM4_ESM.pdf]

Supplementary Fig. 1e

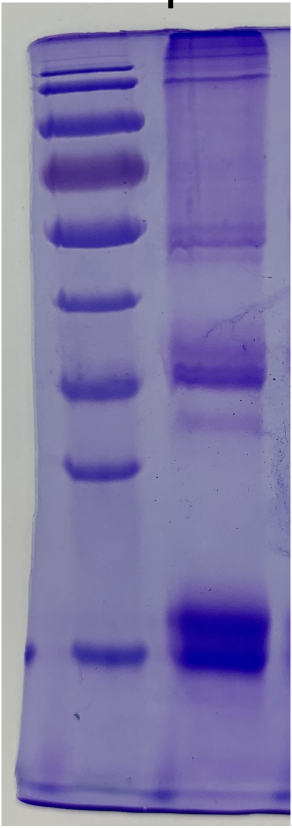

Supplementary Fig. 1f

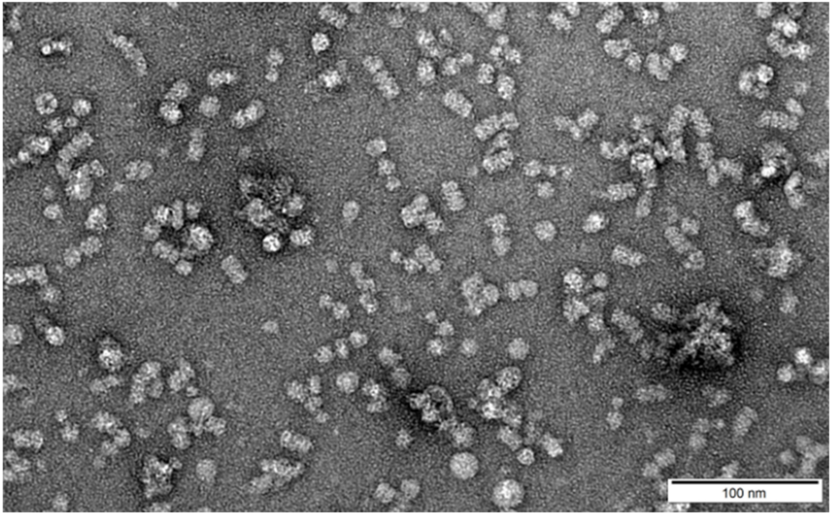

Electron microscopic image of negatively stained CpcL-PBS particles
